# An Ontology-Based Expert System Approach for Hearing Aid Fitting in a Chaotic Environment

**DOI:** 10.3390/audiolres15020039

**Published:** 2025-04-08

**Authors:** Guy Merlin Ngounou, Anne Marie Chana, Bernabé Batchakui, Kevina Anne Nguen, Jean Valentin Fokouo Fogha

**Affiliations:** 1 Department of Computer Engineering, École Nationale Supérieure Polytechnique de Yaoundé, Yaounde P.O. Box 8390, Cameroon; anne.chana@univ-yaounde1.cm (A.M.C.); bernabe.batchakui@univ-yaounde1.cm (B.B.); annekevinanguen091@gmail.com (K.A.N.); 2 Otorhinolaryngology Unit, Bertoua Regional Hospital, Bertoua P.O. Box 40, Cameroon; valentin.fokouo@gmail.com

**Keywords:** hearing aid, personalized fitting, ontology, expert system

## Abstract

Background/Objectives: Hearing aid fitting is critical for hearing loss rehabilitation but involves complex, interdependent parameters, while AI-based technologies offer promise, their reliance on large datasets and cloud infrastructure limits their use in low-resource settings. In such cases, expert knowledge, manufacturer guidelines, and research findings become the primary sources of information. This study introduces DHAFES (Dynamic Hearing Aid Fitting Expert System), a personalized, ontology-based system for hearing aid fitting. Methods: A dataset of common patient complaints was analyzed to identify typical auditory issues. A multilingual self-assessment questionnaire was developed to efficiently collect user-reported complaints. With expert input, complaints were categorized and mapped to corresponding hearing aid solutions. An ontology, the Hearing Aid Fitting Ontology (HAFO), was developed using OWL 2. DHAFES, a decision support system, was then implemented to process inputs and generate fitting recommendations. Results: DHAFES supports 33 core complaint classes and ensures transparency and traceability. It operates offline and remotely, improving accessibility in resource-limited environments. Conclusions: DHAFES is a scalable, explainable, and clinically relevant solution for hearing aid fitting. Its ontology-based design enables adaptation to diverse clinical contexts and provides a foundation for future AI integration.

## 1. Introduction

According to the World Health Organization [1], over 1.5 billion people globally are living with some degree of hearing loss, with projections estimating an increase to 2.5 billion by 2050. Hearing aids are widely recognized as the primary rehabilitation tool for individuals with hearing impairment. As highlighted by Balling et al. [2], the performance of these devices relies significantly on precise and individualized fitting, which typically necessitates repeated adjustments by audiologists in response to user feedback across diverse acoustic settings.

The World Health Organization [1] reports that a shortage of qualified audiologists, particularly in low- and middle-income countries, exacerbates this challenge. Many countries have fewer than one audiologist per million inhabitants, making it nearly impossible to provide adequate follow-up care. In Cameroon, where the prevalence rate of hearing loss is 3.5% according to Wonkam Tingang et al. [3], this disparity is stark, with only four audiologists serving over 27 million people. This shortage results in delayed diagnosis, improper hearing aid fittings, and, often, abandonment of hearing aid use, as reported by Angley et al. [4].

Recent technological advancements have opened up new possibilities for remote and intelligent hearing healthcare delivery, as discussed by Kim et al. [5]. Fabry and Bhowmik [6] demonstrate that artificial intelligence integrated into hearing aids enables improved speech processing and health monitoring via embedded sensors. Similarly, Rapoport et al. [7] discuss how AI systems enhance decision-making in otology and neurotology by facilitating real-time, explainable recommendations.

Despite these advances, artificial intelligence models, particularly those based on deep learning, require extensive training datasets and high computational resources. Fabry and Bhowmik [6] emphasize that these demands limit their deployment in under-resourced environments. In a context characterized by severe data scarcity, dynamic environmental conditions, and continued skepticism toward systems that cannot explain their reasoning, the most reliable resource remains the knowledge of hearing aid experts, as noted by Sutton et al. [8]. These experts often rely on their own experience, manufacturer recommendations, and the scientific literature to fine-tune hearing aids.

Knowledge-based systems provide interpretable, expert-informed reasoning with minimal infrastructure and are particularly valuable in the context of collaborative human–AI interaction for hearing aid personalization. Therefore, this study focuses on the development of an expert system as an intelligent assistant for the adjustment of hearing aids.

Expert systems for hearing aid fitting have been explored for decades. The usual approach consists of determining a vocabulary of terms generally used by wearers to describe their problem and designing a fitting guide that associates each patient’s complaint with appropriate fitting solutions, as outlined by Jenstad et al. [9], Thielemans et al. [10], and Chiriboga [11]. Nevertheless, three major limitations persist in the literature.

**Inconsistent complaint terminology:** The subjective nature of user feedback often lacks the specificity required for audiologists to act upon. For instance, a user’s report of “sound being too loud” may vary depending on the acoustic context, necessitating further clarification and delaying intervention.

**Superficial fitting guides:** Guidelines from organizations such as the American Speech-Language-Hearing Association ASHA [12], the American Academy of Audiology Valente et al. [13], and studies by Chiriboga [11] and Anderson et al. [14] provide foundational recommendations. However, these guidelines tend to be broad, static, and limited to generic suggestions, such as reducing low-frequency gain. They do not provide variations in adjustment parameters necessary to achieve new hearing aid fitting. As hearing aids increasingly incorporate advanced features, the number and complexity of fitting parameters continue to grow, posing challenges even for experienced professionals, as described by Froehlich et al. [15] and Liang et al. [16]. These parameters are also highly interdependent, such that adjusting one can influence the performance of others, as demonstrated by Anderson et al. [14], Anderson et al. [17], and Franck et al. [18]. Consequently, conventional fitting guides often fall short in addressing the nuanced and dynamic requirements of real-world clinical scenarios.

**Absence of open expert systems:** Many hearing aid fitting systems remain proprietary and are not accessible to the broader research and clinical communities. Existing studies, such as those by Jenstad et al. [9], Thielemans et al. [10], and Chiriboga [11], have primarily focused on the knowledge acquisition phase, enabling manufacturers to develop closed expert systems tailored to their own devices. However, given the growing public health impact of hearing loss, the development of open, reusable, and interoperable tools is becoming increasingly important.

Developing an expert system requires choosing an appropriate knowledge representation formalism. Among the available options, ontologies are particularly well suited for medical domains, as they provide a structured way to represent domain-specific concepts and their relationships. Ontologies are widely recognized for their capacity to support semantic interoperability and automated reasoning. As shown by Matentzoglu et al. [19] and Haque et al. [20], ontologies facilitate data integration and decision support across biomedical domains. Furthermore, Rubin et al. [21] argue that ontologies offer the foundational structure necessary for scalable, explainable medical information systems.

In the field of audiology, the work by Dr. C.R. Rene Robin [22] investigated the use of ontologies for cochlear implant recommendations. However, to date, no ontologies have been specifically developed for the representation of knowledge pertaining to hearing aid fitting. Two notable ontologies in related domains include the Hearing Impairment Ontology (HIO), developed by Hotchkiss et al. [23] to model the clinical and genetic aspects of hearing loss, and the Hearing Aid Ontology (HAO), proposed by Napoli-Spatafora [24] for the classification of commercial hearing aids. While HIO lacks technical details regarding device configuration and HAO does not address patient-specific fitting decisions, both ontologies highlight the need for the development of a more comprehensive and clinically relevant ontology specifically tailored to hearing aid fitting.

The objective of this study is to design and validate a dynamic expert system for hearing aid fine-tuning using an ontology-based approach. This system leverages structured knowledge from audiologists, hearing aid manufacturers, and the relevant research literature. Its primary aim is to provide real-time, explainable, and clinically relevant fitting recommendations based on patient-reported complaints.

A case study of this system is carried out on the Balafon hearing aid, developed by the Cameroonian company Bendo (https://www.bendogroup.com/ (accessed on 26 March 2025)).

## 2. Materials and Methods

The following steps outline the procedure for developing an automated system for hearing aid fitting recommendations based on patient complaints:

1. **Data collection and complaint modeling.** Review a dataset of patient complaints to identify common auditory issues. Exclude ambiguous terms and categorize the complaints based on key characteristics.
2. **Development of the self-assessment questionnaire.** Design a self-assessment questionnaire to capture patient complaints.
3. **Design of the fitting guide.** Consult with experts to identify potential hearing aid solutions for each complaint. Classify the complaints into solution-based categories for ease of fitting recommendation.
4. **Construction of the HAFO ontology.** Build the Hearing Aid Fitting Ontology (HAFO) to map patient complaints to hearing aid fitting rules and parameters.
5. **Implementation of the DHAFES system.** Develop the DHAFES system to automatically generate hearing aid fitting recommendations based on the HAFO ontology, allowing audiologists to review and adjust the suggestions.

### 2.1. Data Collection and Complaint Modeling

To model patient complaints effectively, we first conducted a comprehensive review of commonly reported auditory complaints. This involved analyzing (292) complaints documented in previous research studies of Jenstad et al. [9], Chiriboga [11], and the items of the following patient self-evaluation questionnaires: The Abbreviated Profile of Hearing Aid Benefit (APHAB) Cox and Alexander [25], the Hearing Handicap Inventory for the Elderly (HHIE) Newman and Weinstein [26], the Hearing Handicap Inventory for Adults (HHIA) Newman et al. [27], the Glasgow Hearing Aid Benefit Profile (GHABP) Gatehouse [28], the Client Oriented Scale of Improvement (COSI) Laboratories [29], the Speech, Spatial and Qualities of Hearing Scale (SSQ) Laboratories [29], the Structured Hearing Aid Performance Inventory (SHAPI) Schum [30], and the Hearing Needs Assessment and Analysis (HNAA) (https://www.hearinghealthclinic.com/wp/wp-content/uploads/2016/05/HNAAPRIL2015v2.pdf (accessed on 26 March 2025)). A comparative analysis was performed to identify overlapping terms across these sources, prioritizing terms that appeared in multiple references. Redundant terms (e.g., “*painful*” and “*pain*”) were removed to ensure consistency.

A semantic analysis was conducted to categorize ambiguous terms appropriately. For instance, expressions like “*in a barrel*” or *‘‘in a well*” were mapped to categories such as “*echoing sound*” or “*muffled sound*”. Complaints unrelated to auditory perception, such as those referring to the patient’s emotional state, expectations, and cognitive ability, were excluded. Ultimately, a refined list of 53 complaints was established for further characteristic analysis.

**From this analysis**, 6 characteristics of a complaint were identified:

- **Cause**: This presents patients’ difficulty (e.g., *I cannot hear well, I hear a lot of noise, etc.*);
- **Specification**: This indicates if the problem is encountered with particular sounds (e.g., loud sounds or soft sounds) and if so, which ones;
- **Environment**: This indicates the environment in which the problem was encountered *(very quiet, quiet, noisy, and very noisy)*. We made the following considerations according to Paul and association JNA [31]: very quiet (0–20 dB), quiet (25–60 dB), noisy (65–80 dB), very noisy (90–140 dB);
- **Activity**: This indicates the activity the patient was engaged in when he/she encountered the problem (e.g., *conversation with someone/in a group/on the phone, etc.*);
- **Frequency**: This indicates the number of times the patient encountered the problem (*rarely, occasionally, often, usually, almost always, always*);
- **Discomfort level**: This indicates the degree to which this problem bothers the patient (*tolerable, annoying, unbearable*).

### 2.2. Development of the Self-Questionnaire

A structured self-assessment questionnaire was developed based on the complaint model and derived from the most common questionnaires in the literature (Cox and Alexander [25], Newman and Weinstein [26], Newman et al. [27], Gatehouse [28], Laboratories [29], Schum [30], Gatehouse and Noble [32]). It consists of six closed-ended questions, each corresponding to one of the six characteristics of the complaint.

The questionnaire is divided into three sections: the **nature of the complaint** section captures cause and specificity. The **listening context** section captures environment and activity. The **impact assessment** section captures frequency and degree of discomfort. Each complaint characteristic includes a set of predefined items (cause: 18; specificity: 7; environment: 5; activity: 8; frequency: 7; discomfort level: 3). These combinations yield 5040 distinct complaints, or 125,840 when frequency and discomfort are considered. By selecting responses for each question, patients generate a structured description of their auditory complaint. Complaints were numerically encoded to enable systematic analysis, and anomalous responses were filtered based on logical inconsistencies (e.g., “*My voice is noisy when I listen to the radio in a quiet room*”).

The questionnaire was implemented as a digital interface while preserving the psychometric properties of the original instruments. To improve accessibility, the language was simplified without altering meaning. Patients responded by using touchscreen-compatible rating scales, with questions displayed one at a time to minimize cognitive load along with clear instructions and examples.

Its development followed a rigorous multistep process to ensure comprehensive coverage of patient-reported hearing aid issues. The questionnaire underwent expert validation, with audiologists refining its clarity, relevance, and precision for complaint classification, resulting in a robust tool for real-time hearing aid fitting assessment.

To support diverse populations, the questionnaire is available in English and French, with translations reviewed by native-speaking audiologists. Since responses are encoded numerically, they remain language-independent, ensuring seamless multilingual integration. This approach also accounts for differences in audiological terminology and how users describe hearing complaints.

For the full questionnaire, see Appendix A.

### 2.3. Design of the Fitting Guide

The development of the fitting guide include knowledge from experienced hearing care professionals, manufacturer’s recommendations, and research findings.

1. **Experts’ hearing care experiences.**
  Complaint modeling according to 6 characteristics allows reporting 5040 different complaints. We asked hearing care professionals to select among these the coherent complaints commonly reported by wearers and to associate fitting solutions. Based on expert consultations, we classified these solutions into six categories:
  - **Recommendation (R)**: This tells the patients what they can do to fix or alleviate the situation. For example, increasing or decreasing the volume of the hearing aid;
  - **Physical adjustment (AP)**: This includes situations when the complaint refers to a change in the characteristics or physical elements of the prosthesis. For example, changing the tube, cleaning the prosthesis;
  - **Electroacoustic adjustment (AE)**: This solution requires the knowledge of the parameter to be modified and the operation to be applied to this parameter (increase the global gain, decrease the low-frequency gain, etc);
  - **Therapy (T)**: It is used when the complaint refers to an intrinsic characteristic of the patient, such as speech comprehension problems like “*I hear but I do not understand*”;
  - **Consultation (C)**: This occurs when the information contained in the complaint is not sufficient to adjust;
  - **Accommodation (AC)**: This situation arises when no immediate solution can be found and the patient must adapt to it. As noted by AuditionSanté [33], the use of hearing aids may require an adjustment period.
  Audiologists were instructed to prioritize solutions for each complaint. In clinical settings, they would attempt the first solution, and if the patient remained dissatisfied, they would proceed to the next solution, continuing this process until the patient was satisfied. Complaints with identical solutions were grouped into an equivalence class, while unresolved complaints were categorized into a “consultation” class. For electroacoustic adjustments, audiologists were also asked to specify which fitting parameters to modify, such as frequency response, compression characteristics, or noise reduction. Following this procedure, 33 complaint classes were identified, each associated with a specific solution. This forms the basis of our fitting guide, an excerpt of which is presented in Table 1. For the full fit guide, see Appendix B.
  Based on the developed guide, the field labeled *Command* contains the values **decrease −** and **increase +**, indicating the necessary operations for adjusting the fitting. For example, for the class described as “*loud sound in a quiet environment*” (see Table 1, line 11), the corresponding solution is “*decrease MCL*”. However, in order to decrease the gain, it is important to consider the specific constraints of the hearing aid (e.g., the maximum and minimum values) and the extent to which the gain should be adjusted to achieve a noticeable change. While the first piece of information is provided by the manufacturer, the last one can be provided by the hearing care professional’s experiences and/or relevant scientific research.
2. **Manufacturer’s recommendations.**
  The manufacturers of the Balafon hearing aid have provided detailed recommendations for each hearing aid parameter, including default values, critical values, constraints, recommended variations, and relevant use cases. For instance, the permissible range for MCL (Most Comfortable Loudness) is 0–70 dB. Figure A2b,d, in Appendix C.2 describe how this manufacturer’s information is used.
3. **Research results.**
  A review of the literature was conducted to identify studies addressing hearing aid parameter adjustment methods. This initial investigation focused on works related to frequency gain, as reported by Caswell-Midwinter and Whitmer [34], Jenstad et al. [35], Dirks et al. [36], and Caswell-Midwinter and Whitmer [37], as well as studies on dynamic compression by Franck et al. [18] and Anderson et al. [17]. Each study was classified according to the relevant parameter, experimental conditions, and reported outcomes, such as recommended adjustments.
  Based on the findings, recommended adjustments for a noticeable frequency gain include variation in a range of 4–12 dB. Depending on the clinical context, the applied gain may correspond to the minimum, maximum, or average of these values. For example, a solution for the complaint “*loud sound in quiet environment*” may involve “*decrease MCL by 6 dB*”. These findings were integrated into HAFO (available at https://github.com/annekevs/hafo (accessed on 26 March 2025)), the domain ontology developed for hearing aid fitting.

### 2.4. Construction of the HAFO Ontology

After modeling the complaints and developing the fitting guide, a structured system is needed to interpret the complaint vocabulary and infer appropriate data based on the rules outlined in the fitting guide. The Hearing Aid Fitting Ontology (HAFO) was designed to serve this purpose, providing a standardized vocabulary that enables prediction hearing aid settings by linking patient complaints to the fitting rules.

**Figure 1:**
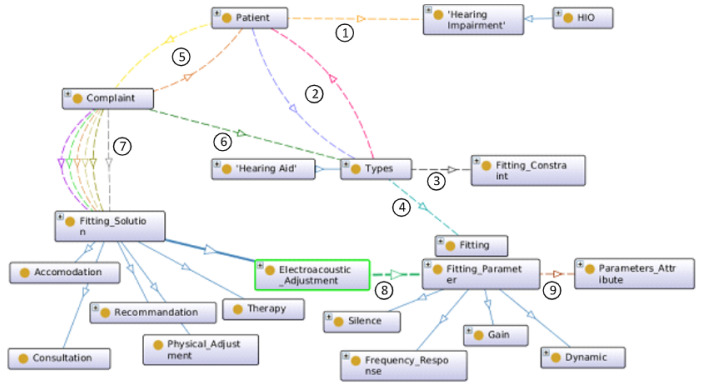
Minimalist graph of HAFO ontology.

1. **HAFO design.**
  The concepts related to hearing aid fitting mainly involve the patient, the complaint, and the fitting parameters of the hearing aid to be modified. The domain knowledge of the hearing impairment is based on the Hearing Impairment Ontology developed by Hotchkiss et al. [23], while the commercial description of the hearing aid is provided by in Hearing Aid Ontology designed by Napoli-Spatafora [24]. Figure 1 gives a graphical overview of the concepts related to the fitting of hearing aids. A patient has ① *hearing loss* and uses a hearing aid as ② treatment. Each hearing aid has ③ fitting constraints. For example, body hearing aids (traditional large hearing aids worn on the body) have a wider range of amplification than Behind-The-Air (BTE) aids. A hearing aid type has ④ a current fitting. A patient submits ⑤ a complaint. A complaint is characterized by the following: description, cause, specificity, environment, activity, frequency, and degree of discomfort. A complaint is related to ⑥ a hearing aid type. Each patient complaint is associated with one or more ⑦ fitting solutions (consultation, recommendation, physical adjustment, therapy, accommodation, and electroacoustic adjustment) depending on their degree of priority. The electroacoustic adjustment consists of a modification of the ⑧ adjustment parameters (or electroacoustic characteristics) of the hearing aid. Each parameter has its own ⑨ specific attributes (value range, mode, default value, unit, etc.).
2. **HAFO implementation.**
  The HAFO ontology was developed in English using OWL 2 to define concepts and relationships for interoperability. Protégé (http://protege.stanford.edu (accessed on 26 March 2025)) 5.5.0 facilitated the ontology creation, while SWRL enabled rule-based logic based on the fit guide in Section 2.3. The inference engine Pellet (https://www.w3.org/2001/sw/wiki/Pellet (accessed on 26 March 2025)) was employed to infer implicit relationships. Pellet performs reasoning tasks such as consistency checking, classification, and query answering by processing the ontology’s defined relationships. This ensures that the system’s recommendations are logically consistent with HAFO, supporting dynamic adaptation to various fitting scenarios. For instance, Pellet can suggest adjustments like increasing microphone sensitivity or enhancing noise reduction for complaints about hearing in noise. OntoGraf was used for visualizing the ontology structure.

### 2.5. DHAFES System Architecture

**Figure 2:**
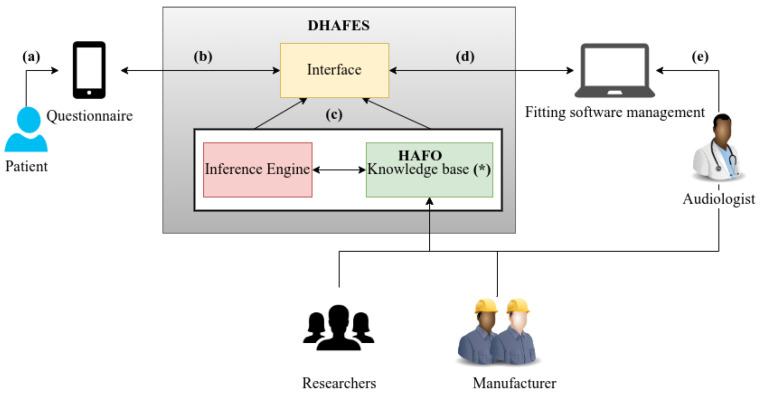
DHAFES expert system functional architecture. (a) The patient complains by answering the questionnaire. (b) DHAFES interacts with m-Health application to retrieve the patient’s complaints/send back the fitting recommendation. (c) DHAFES infers from the knowledge base and generates a fitting recommendation for the complaint. (d) DHAFES interacts with the fitting software to send the recommendation/obtain feedback from the audiologist. (e) The audiologist can approve, modify or reject the DHAFES recommendation.

DHAFES is a practical implementation of the HAFO ontology, designed to predict hearing aid adjustment solutions based on a patient’s specific complaint. The system architecture of DHAFES, presented in Figure 2, includes an inference engine (Pellet), an API for integration with existing (tele)fitting software, and a knowledge base composed of HAFO-defined classes, instances, and SWRL (Semantic Web Rule Language) rules derived from the fitting guide. The expert system DHAFES was implemented as a RESTful API to enable communication with existing fitting software. The system was deployed on a Tomcat server and uses the Java libraries OWL API and Openllet-OWLAPI for settings prediction. The HAFO ontology is stored as an OWL file on the server and contains the necessary information for predicting a configuration for the hearing aid.

The system is built to operate efficiently in both clinical and remote settings, with or without direct audiologist involvement. The workflow follows four key phases: user input, system reasoning, expert validation, and remote fitting.

1. **Step 1: Patient interaction and complaint collection.**
  Patients interact with DHAFES through a bilingual mobile interface, completing the structured, expert-validated questionnaire designed in Section 2.2.
2. **Step 2: Ontology-based inference and recommendation generation.**
  Once the questionnaire is submitted, DHAFES converts the patient’s structured responses into an ontological query, uses a semantic reasoning engine (Pellet) in combination with the HAFO ontology, determines which class(es) of complaints the input falls under (e.g., feedback, speech-in-noise, volume discomfort), and applies predefined expert rules to generate recommendations and parameter adjustments. Each complaint class is mapped to a specific response strategy, such as electroacoustic adjustment (e.g., reduce gain at certain frequencies), physical adjustment (e.g., change ear tip), recommendation (e.g., better placement of the hearing aid), or referral or therapy (e.g., auditory training or ENT consultation).
3. **Step 3: Audiologist review and remote fitting.**
  All recommendations are reviewed by an audiologist via the Fitting Management Software interface. The audiologist can approve, modify, request more information, or suggest clinical referral. Once approved, adjustments are applied to the hearing aid, including in remote settings, ensuring clinical oversight. After the adjustment, the patient is prompted to rate their satisfaction during follow-up. These feedback points are logged to track resolution rates per complaint class, flag complaints needing ontology refinement, and support continuous improvement of DHAFES rules via expert review.
4. **Step 4: Feedback and system evolution.**
  Post-adjustment, patients can rate their satisfaction. Expert analysis of this feedback informs updates to the HAFO ontology. Although DHAFES is not self-learning, it evolves through expert input, clinical collaboration, manufacturer guidance, and research. The system is designed to be robust, explainable, and operable without constant internet access or local audiology services.

## 3. Results

**Figure 3:**
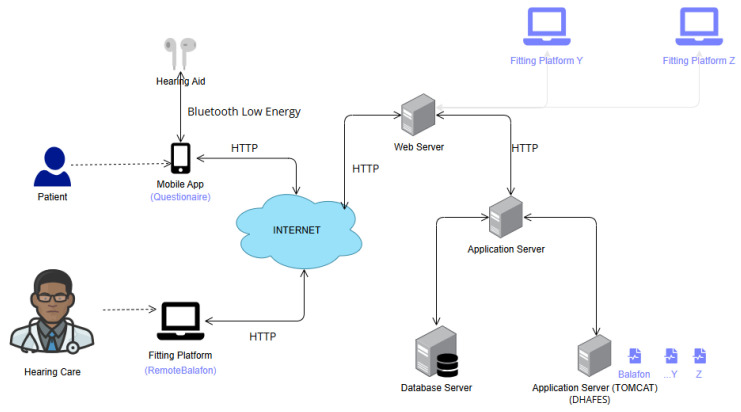
Integration of DHAFES with RemoteBalafon.

DHAFES is designed to be manufacturer-agnostic and can serve as an intelligent assistant for hearing aid adjustments based on patient complaints. The following case study illustrates the use of DHAFES within the context of Balafon hearing aids, developed by the company Bendo [38]. To achieve that, the following steps were followed:

1. **Step 1: HAFO verification.**
  This step aims to verify that the ontology includes all the necessary parameters for the Balafon hearing aid. If any are missing, new concepts must be added. For example, one of the features of the Balafon hearing aid is the silence detection, which is managed by the parameter enerFaibl. So, we added the “Silence” class as a subclass of the “Fitting Parameter” class of the ontology (see Figure A2b in Appendix C.2).
2. **Step 2: Instances creation.**
  In this step, we create permanent instances that will be used by the inference engine to process the query. These instances involve hearing aid models (BTE, body hearing aid), parameters, adjustment constraints (related to device limitation), etc.… For instance, the BTE model of the Balafon hearing aid limits MCL to 70 dB despite the typical 0–120 dB range (see Figure A2d in Appendix C.2). Such constraints ensure that recommendations remain within device capabilities. Other instances related to Patient, Hearing Impairment, Hearing Aid, Complaint, and Fitting can be temporary and will be created dynamically during the prediction.
3. **Step 3: Rules update.**
  If necessary, adjustment rules (derived from the fit guide developed in Section 2.3) can be updated based on the patient’s complaints, parameter ranges, and adjustment constraints. An example of a rule, written in SWRL language, is provided in Appendix C.3. Additional rules can be added based on patient characteristics such as age, gender, and demographics, with the system automatically inferring the appropriate adjustments. The result of this step is HAFO-Balafon, a fully customized version of HAFO for the hearing aid Balafon. This knowledge base will be stored in DHAFES, allowing the RemoteBalafon Platform to make API calls for adjustment recommendations.
4. **Step 4: Integration.**
  The DHAFES expert system was integrated with the existing remote fitting platform called RemoteBalafon, and the resulting physical architecture is shown in Figure 3. The digital questionnaire is integrated into the m-Health application on the patient side. When a patient reports a complaint, the encoded data are transmitted to both the audiologist’s web platform and the DHAFES system. DHAFES then performs inference and returns the recommended adjustments along with an explanation of the reasoning behind them to the audiologist.

In the same way, DHAFES can be used by any hearing aid manufacturer and provides a result in near real time. For further details, refer to the Appendix C and Appendix D.

## 4. Discussion

### 4.1. Strengths and Clinical Impact

**Multilanguage support.** One of the key advantages of the DHAFES system is its multilingual support, which enhances its accessibility and usability across diverse patient populations. The system is designed to accommodate multiple languages, allowing users from various linguistic backgrounds to benefit from the hearing aid fitting process. This multilingual capability is made possible due to the encoded nature of the questionnaires used. By supporting a range of languages, DHAFES can help bridge language barriers, making hearing care more inclusive and accessible for patients worldwide.

**Figure A1:**
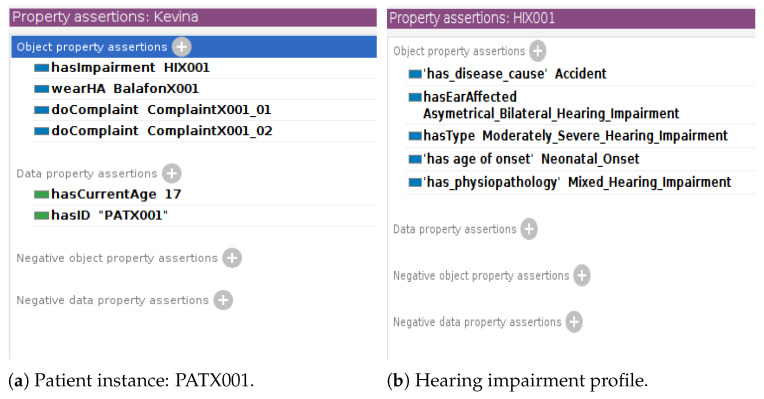
(**a**) Representation of patient instance PATX001. (**b**) Associated hearing impairment profile used in DHAFES for personalized fitting recommendations.

**Figure A2:**
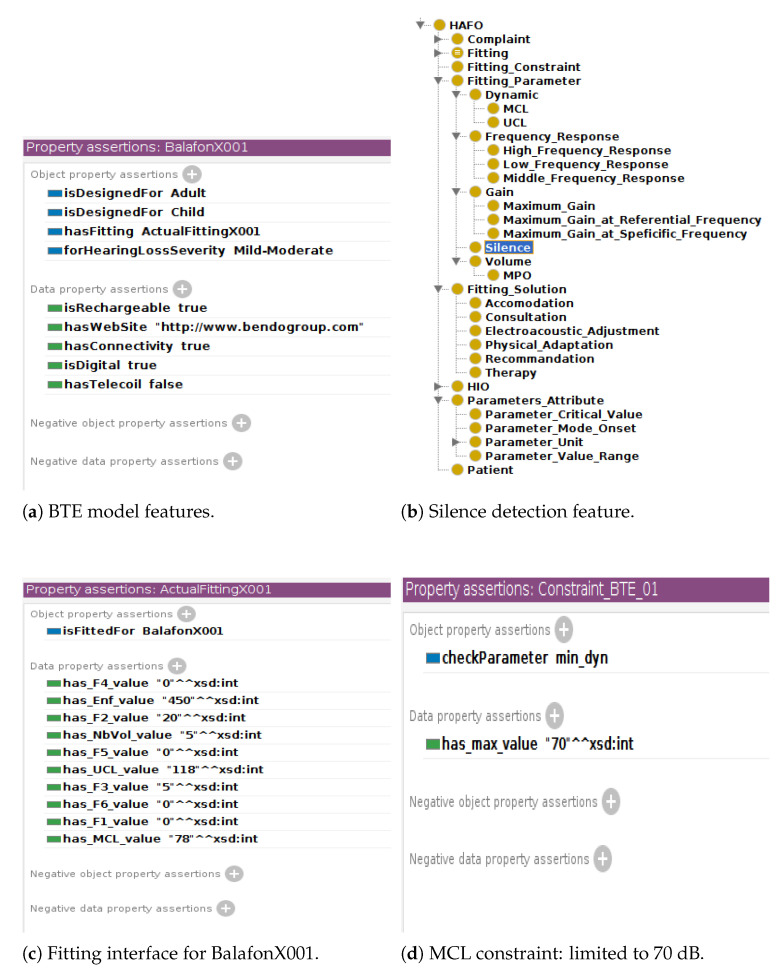
Manufacturer’s details: (**a**) BTE Hearing Model worn by patient PATX001. (**b**) Silence Detection Feature added as fitting parameter. (**c**) Actual fitting of the hearing aid BalafonX001 for Patient PATX001. (**d**) Adjustment constraint of the parameter min_dyn (MCL).

**Individual variability.** The HAFO ontology integrates multiple dimensions of patient context, including demographic characteristics (such as age and gender), hearing aid model, prescribed fitting method (e.g., NAL-NL2, DSL), and relevant comorbidities (such as cognitive decline or diabetes), by defining them as subclasses or conditions within the ontology. This structure enables DHAFES to apply differentiated fitting strategies when corresponding expert rules are available. For example, the system can distinguish that a 70-year-old patient with cognitive impairment may require a different gain adjustment or compression strategy than a 30-year-old patient with the same audiometric profile but no cognitive limitations. This capability is already embedded in the system design; the primary constraint lies in the availability of clinically validated rules to define such variations. As new clinical guidelines and research findings become available, they can be incorporated into the system without requiring changes to its core architecture. An example is provided in Figure A1a (Appendix C.1).

**Educational utility.** In addition to its role in clinical decision-making, DHAFES serves as a valuable training tool. Given the global shortage of qualified audiologists, especially in low-income countries, DHAFES can be used as an intelligent tutoring system to provide a sustainable way to scale hearing care education. Trainees can simulate real-world complaint scenarios and receive structured guidance on optimal responses. Novice practitioners can use it to understand expert-level fitting strategies. By simulating common complaints and demonstrating reasoning pathways, DHAFES can shorten the learning curve for new professionals and reduce reliance on expensive international training programs. Thus, DHAFES not only automates personalized adjustment but also aims to actively contribute to building human expertise.

**Interoperability.** The modular and brand-agnostic nature of HAFO enables interoperability across various hearing aid platforms. DHAFES features a flexible interface that enables manufacturers to define their devices and incorporate new technologies without necessitating extensive retraining. Manufacturers can update the ontology with proprietary parameters while still benefiting from a shared reasoning framework. This not only reduces development overhead but also enhances consistency in patient care. This design facilitates system adoption and enables clinicians to use a standardized platform across various devices, thereby enhancing efficiency and reducing on-boarding time for new professionals.

**Integration with existing system technology.** The DHAFES system can be valuable not only in the absence of an audiologist but also in scenarios where audiologists are accessible. Specifically, the system could serve as an additional tool to enhance the efficiency of audiologists by providing immediate adjustments or as a first step in the hearing aid fitting process, potentially reducing the time required for consultations. Furthermore, DHAFES could be beneficial for patients in remote areas or those with mobility issues, providing them with a level of independence while waiting for professional follow-up.

### 4.2. DHAFES vs. AI/ML Models

**Explainability and transparency.** AI systems integrated into digital signal processors (DSPs) frequently function as opaque black boxes, providing limited insight into the rationale underlying parameter adjustments. In contrast, DHAFES is founded on a rule-based expert system anchored in a clinical ontology (HAFO), where each decision is explicitly linked to clinical reasoning. This level of interpretability is critical for clinician trust, particularly in medical domains where audibility and effective patient communication are essential, as emphasized by Sadeghi et al. [39].

**Suitability for low-resource settings.** Many AI-enabled hearing aids depend on continuous data logging, computationally intensive processing, and often cloud connectivity to perform effectively. DHAFES, by design, operates offline and without reliance on user data, making it especially suitable for resource-constrained environments such as rural or underserved regions where infrastructure and professional support may be limited or inconsistent. This approach aligns with findings from Tagne et al. [40], who highlight the importance of accessibility and acceptability of e-health solutions in areas with limited resources.

**Integration of clinical judgment.** Although AI systems can adapt to various soundscapes and usage patterns, they typically lack the depth of clinical context. DHAFES extends beyond electroacoustic adjustments by incorporating broader clinical interventions such as therapy referrals, user education, and physical device inspection. It encapsulates the decision-making logic of experienced audiologists, particularly in cases where acoustic modifications alone are insufficient.

**Complementary design philosophy.** DHAFES is not intended to replace DSP-integrated AI systems but rather to function as a complementary tool. In advanced clinical environments, it can validate, explain, or override AI-based recommendations when inconsistencies arise. In lower-resource contexts, it can independently guide fittings with minimal technological requirements, thereby bridging the gap between expert clinical practice and accessible digital solutions. This synergy provides both adaptability and control, enhancing user satisfaction.

### 4.3. Limitations and Future Work

**Bias on patient self-reporting.** Despite its advantages, DHAFES faces several challenges. The most prominent is the subjectivity of patient self-reporting. As mentioned by Chodosh and Blustein [41], variations in how users describe auditory experiences can lead to inaccuracies. Although DHAFES employs structured prompts and predefined symptom categories to minimize ambiguity and improve consistency, its reliance on patient self-reporting remains a fundamental limitation, potentially introducing bias. Future work will explore integrating self-reported data with objective measurements and periodic clinical input to improve the accuracy of hearing aid fittings and reduce dependency on potentially inconsistent patient input.

**Absence of objective measurements.** Another limitation is that DHAFES cannot directly measure real-time hearing thresholds or gain or simulate free-field audiometry. As investigated by Sandström et al. [42], technologies such as smartphone-integrated audiometry or calibrated external microphones may enhance this capability in future versions.

**Missing automatic updates.** DHAFES also lacks probabilistic learning capabilities. Unlike ML systems, it does not refine its rules automatically over time. Integrating hybrid AI–ontology models could provide adaptive learning while preserving interpretability.

## 5. Conclusions

The fitting of a hearing aid is a crucial part of the patient’s hearing rehabilitation. This article presents an expert system based on an ontology that integrates knowledge from hearing aid practitioners, manufacturers, and researchers to improve fitting accuracy. The system offers personalized adjustment recommendations based on the patient’s specific listening situations.

This work contributes to (1) audiology, (2) biomedical ontologies, and (3) the hearing aid industry. In audiology, six key complaint characteristics are proposed to ensure consistent patient-reported complaints. In biomedical ontologies, HAFO, the first ontology for hearing aid fitting, is introduced, with potential for use in both fitting recommendation systems and training tools for practitioners. For the hearing aid industry, the DHAFES expert system provides personalized adjustments based on patient characteristics and listening environments, offering explainable reasoning grounded in clinical expertise. It can also be used as an educational tool for training audiologists.

The system produces a comprehensive adjustment guide, enabling context-appropriate adjustments in near real time. The HAFO ontology can be enhanced with additional expert knowledge. While AI-powered hearing aids are revolutionizing the field of audiology, DHAFES fills essential gaps that current DSP-based AI cannot address alone—notably in transparency, clinical generalization, low-resource adaptability, training scalability, and cross-brand integration.

This work demonstrates the potential of using ontologies for intelligent hearing aid fitting systems. Future developments should focus on clinical validation to assess performance, ensuring that DHAFES effectively improves user outcomes and is capable of safely handling growing amounts of data while maintaining privacy and data integrity.

## Figures and Tables

**Table 1:** An extract of the fitting guide.

N°	Complaint	Solution	Category ^1^	Parameter ^2^	Command	Recommendation
1	not hearing sounds	C				The hearing aid may be in standby mode. Restart (turn off and on) the hearing aid and check the battery level. Ensure that your hearing aid is on.
2	wind noise in quiet environment	EA	dynamic	MCL	decrease−	Do not worry, this is the intrinsic noise of the hearing aid.
3	whistling in quiet environment	AP				Check that your hearing aid is properly positioned in the ear.
4	whistling in noisy environment	AP				Check that your hearing aid is properly positioned.
5	whistling	EA	dynamic	UCL	decrease−	Lower the volume.
6	noise in quiet environment	EA	dynamic	MCL	decrease−	Lower the volume.
7	noise in noisy environment	EA	volume	nb_vol	increase+	Lower the volume.
8	hearing but not understanding	T				Follow a therapy. Please make an appointment with an audiologist.
9	loud sounds	EA	dynamic	UCL	decrease−	Lower the volume.
10	soft sounds loud in quiet environment	EA	dynamic	MCL	decrease−	Lower the volume.
11	loud sound in quiet environment	EA	dynamic	UCL	decrease−	Lower the volume.
12	loud sound in noisy environment	C				Lower the volume, reduce sensitivity.
13	loud sound	EA	dynamic	MCL	decrease−−	Lower the volume.
14	loud soft sounds in quiet environment	EA	dynamic	UCL	increase++	Increase the volume.
15	loud soft sounds in noisy environment	EA	dynamic	MCL	decrease−−	
16	loud soft sounds	EA	dynamic	UCL	increase++	Increase the volume.
17	soft sounds soft in quiet environment	EA	dynamic	MCL	increase+	Increase the volume.
19	soft sounds soft	EA	dynamic	MCL	increase+	Increase the volume.
20	soft sound in quiet environment	EA	dynamic	MCL	increase++	
23	sound cutting in quiet environment	EA	silence	enerFaibl	decrease−−	
24	sound cutting in noisy environment	EA	silence	enerFaibl	decrease−−−−	
25	sound cutting	EA	silence	enerFaibl	decrease−−	

^1^ depends on manufacturer; ^2^ not depending on the manufacturer. Note: accommodation (AC), physical adjustment (AP), electroacoustic adjustment (EA), consultation (C), recommendation (R), therapy (T). Balafon parameters: MCL (Most Comfortable Loudness) named min_dyn, UCL (Uncomfortable Loudness Level) named max_dyn, nb_vol, enerFaibl.

**Figure A3:**
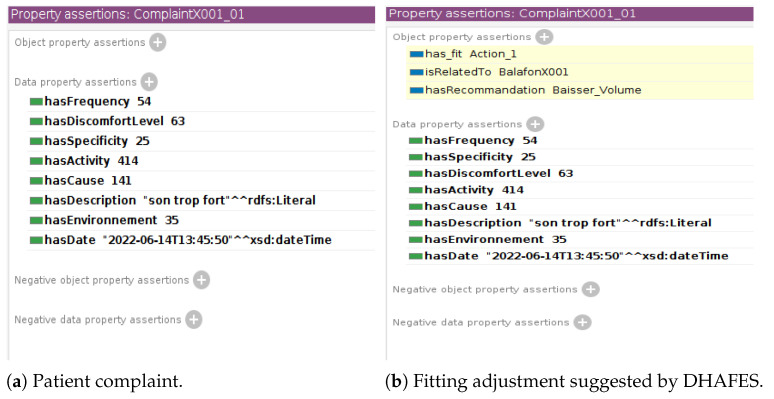
DHAFES inference process: (**a**) The patient reports an issue with loud sounds in quiet environments. (**b**) The system recommends reducing the MCL parameter by 6 dB.

**Figure A4:**
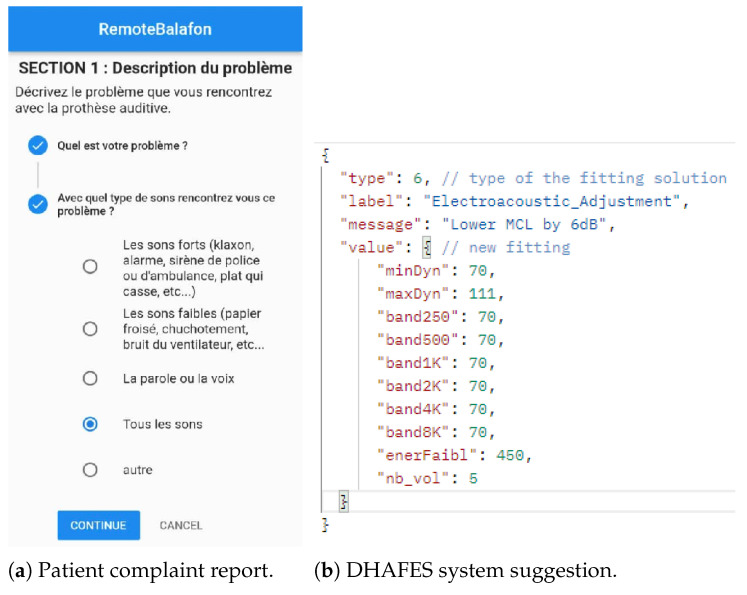
Graphical representation of the patient complaint handling process in DHAFES. (**a**) A patient submits a complaint report. (**b**) The DHAFES system provides a suggested solution based on the complaint.

**Figure A5:**
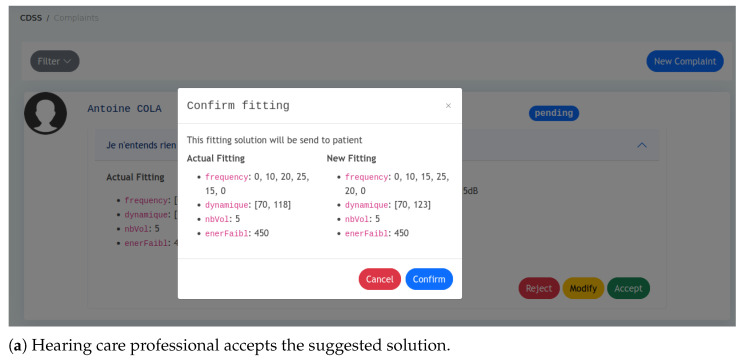

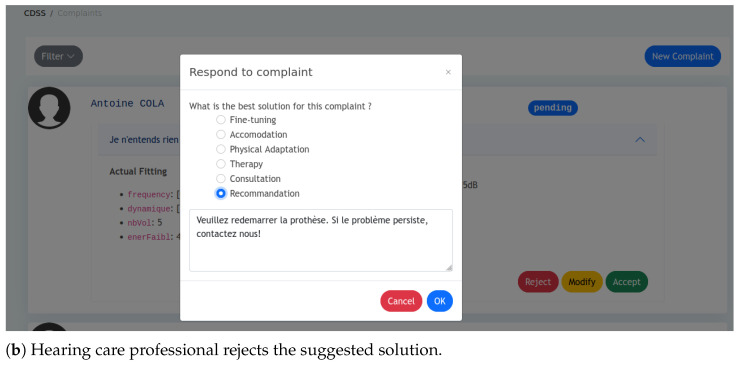
Graphical representation of the patient complaint handling process in DHAFES. (**a**) The hearing care professional accepts the DHAFES suggestion. (**b**) The hearing care professional rejects the DHAFES suggestion.

## Data Availability

The original contributions presented in the study are included in the article, further inquiries can be directed to the corresponding author.

## Appendix A. Self-Evaluation Questionnaire

This questionnaire allows you to describe the problem you are experiencing with your hearing aid.

**Instructions:** Complete the sentences, and if you do not find an appropriate answer, you can add your own under “Other.’ Questions marked with (*) are optional.

### Appendix A.1. Section 1: Describe the Problem You Are Facing

The problem I am experiencing is as follows:

- I do not hear anything.
- I hear a lot of noise:
  - The sound of rain or wind.
  - Ambient noises.
  - Buzzing, grinding noises.
  - Whistling sounds.
  - Other:__________________________
- I hear but do not understand.
- I do not hear well because:
  - The sound is too loud.
  - The sound is too weak.
  - The sound cuts out intermittently.
  - The sound is strange, unnatural.
  - The sound is distorted.
  - The sound is muffled.
  - The sound is metallic.
  - The sound is painful.
  - Other:__________________________
- I hear an echo.
- Other:__________________________

(*) This generally happens with specific sounds such as:

- Loud sounds (horn, alarm, siren, dish breaking, running water, door slamming, etc.).
- Soft sounds (paper rustling, fan noise, etc.).
- Speech or voice.
- My own voice.
- Men’s voices (deep pitch).
- Women’s or children’s voices (high pitch).
- Any type of voice.
- All sounds.
- Other:__________________________

### Appendix A.2. Section 2: Describe the Situation Where You Experienced This Problem

When I am in an environment that is:

- Very quiet (recording studio, etc.).
- Quiet (residential area, office, etc.).
- Noisy (playground, restaurant, traffic, party, etc.).
- Very noisy (bar, market, concert, packed stadium, etc.).
- Any environment.
- Other:__________________________

While:

- Talking.
- Talking to someone.
- Talking in a group.
- Talking on the phone.
- Listening to the radio, TV, or a movie.
- Attending a class, conference, or seminar
- Listening to music.
- Doing anything.
- Other:__________________________

### Appendix A.3. Section 3: Frequency and Discomfort Level

This happens:

- Never.
- Rarely.
- Occasionally.
- Sometimes.
- Often.
- Very often.
- Always.

Furthermore, I find it:

- Bearable.
- Annoying.
- Unbearable.

### Appendix A.4. Summary: Complaint Description

Based on patient input, the following are examples of a complaint’s description that will be generated. Then the patient is asked to confirm whether this prompt describe exactly their problem. The patient can also add additional information that may be more personal. The first sample presents a coherent complaint and the second one an incoherent complaint. During the answering process, caution has been taken so that the patient cannot report such an incoherent complaint.

- Sample 1 (coherent):
  *When I am in a **quiet place**, I have difficulty **conversing with someone** because **the sound cuts out**. I experience this problem **all the time**, and **I find it unbearable**.*
- Sample 2 (incoherent):
  *When I’m in a **quiet place,** **listening to the radio**, **my voice** makes **a lot of noise**.*

Does this description correspond to the problem you’re facing?

- Yes.
- Almost.
- No.

*Please provide here all additional information you want your hearing care provider to know.* _____________________________________________________________________________________________________________________________________________________________________________________________

## Appendix B. Fitting Guide

The fitting guide, presented in Table A1, outlines 33 primary complaint classes commonly reported by hearing aid users along with their corresponding fitting solutions. The **Parameters** column contains brand-specific adjustments, tailored in this case to the Balafon hearing aid from Bendo.

**Table A1:** Fitting guide.

N°	Complaint	Solution ^*a*^	Category ^*b*^	Parameter ^*c*^	Command	Recommendation
1	not hearing sounds	C				The hearing aid may be in standby mode. Restart (turn off and on) the hearing aid and check the battery level. Ensure that your hearing aid is on.
2	wind noise in quiet environment	EA	dynamic	MCL	decrease−	Do not worry, this is the intrinsic noise of the hearing aid.
3	whistling in quiet environment	AP				Check that your hearing aid is properly positioned in the ear.
4	whistling in noisy environment	AP				Check that your hearing aid is properly positioned.
5	whistling	EA	dynamic	UCL	decrease−	Lower the volume.
6	noise in quiet environment	EA	dynamic	MCL	decrease−	Lower the volume.
7	noise in noisy environment	EA	volume	nb_vol	increase+	Lower the volume.
8	hearing but not understanding	T				Follow a therapy. Please make an appointment with an audiologist.
9	loud sounds are very loud	EA	dynamic	UCL	decrease−	Lower the volume.
10	soft sounds loud in quiet environment	EA	dynamic	MCL	decrease−	Lower the volume.
11	loud sound in quiet environment	EA	dynamic	UCL	decrease−	Lower the volume.
12	loud sound in noisy environment	C				Lower the volume, reduce sensitivity.
13	loud sound	EA	dynamic	MCL	decrease−−	Lower the volume.
14	loud sounds are faint in a quiet environment	EA	dynamic	UCL	increase++	Increase the volume.
15	loud sounds are faint in noisy environment	EA	dynamic	MCL	decrease−−	
16	loud sounds are soft	EA	dynamic	UCL	increase++	Increase the volume.
17	weak sounds are soft in quiet environment	EA	dynamic	MCL	increase+	Increase the volume.
18	weak sounds are soft in noisy environment	R				Ask the speaker to speak louder.
19	weak sounds are very soft	EA	dynamic	MCL	increase+	Increase the volume.
20	soft sound in quiet environment	EA	dynamic	MCL	increase++	
21	soft sound in noisy environment	R				Lower the volume, ask the speaker to speak louder.
22	soft sound	EA	dynamic	MCL	increase++	
23	sound cutting in quiet environment	EA	silence	enerFaibl	decrease−−	
24	sound cutting in noisy environment	EA	silence	enerFaibl	decrease−−−−	
25	sound cutting	EA	silence	enerFaibl	decrease−−	
26	distorted sound	EA	frequency	medium		Make speech intelligible.
27	muffled sound	EA	frequency	low and medium		Attenuate low frequencies, amplify mid frequencies.
28	painful sound ( high-pitched sound)	EA	frequency	high	decrease−	
29	loud male voices in quiet environment	EA	frequency	low	decrease−	
30	loud children’s voices in quiet environment	EA	frequency	high	decrease−	
31	weak sounds are loud	EA	dynamic	MCL	decrease−	Lower the volume.
32	I hear but I do not understand + specific voice	C				
33	other	C				

^*a*^ Solution: accommodation (AC), physical adjustment (AP), electroacoustic adjustment (EA), consultation (C), recommendation (R), therapy (T); ^*b*^ independent of the manufacturer; ^*c*^ dependent on the manufacturer.

- **MCL (Most Comfortable Level):** The volume level at which sound is perceived as comfortable for listening.
- **UCL (Uncomfortable Loudness Level):** The threshold at which sound becomes intolerably loud.
- **Frequency:** This governs the adjustment of sound pitch, affecting the amplification of low, mid, or high frequencies to enhance speech clarity and listening comfort.
- **enerFaibl:** A parameter regulating silence detection.
- **nb_vol:** A control for adjusting the hearing aid’s overall volume output.

This guide serves as a reference for optimizing hearing aid settings based on user feedback, ensuring both comfort and clarity in various listening environments.

## Appendix C. DHAFES Entities

In this section, we present some entities of the DHAFES.

### Appendix C.1. HAFO Patient’s Characteristics

DHAFES incorporates patient properties to describe individual characteristics while also considering specific hearing impairments. Importantly, these properties do not contain personal data, ensuring strict user privacy protection. Figure A1b illustrates an instance of patient PATX001, a 17-year old suffering from a moderately severe hearing impairment (HIX001).

By integrating such detailed patient descriptions, DHAFES accounts for patient variability. For example, a 70-year-old patient with cognitive impairment and mild sensorineural hearing loss may require a different gain adjustment or compression strategy than a 30-year-old patient with the same audiogram but a different cognitive profile. This personalized approach enhances the accuracy of hearing aid fitting recommendations.

### Appendix C.2. Manufacturer’s Brand

Hearing aid manufacturers develop various models with distinct features, shapes, and parameter constraints to accommodate different patient needs. DHAFES ensures that each manufacturer can define the specific functionalities available in their models and describe how fitting parameters should be managed.

For instance, Figure A2 depicts the hearing model BTE worn by patient PATX001. This model includes the silence detection feature, is fitted with ActualFittingX001 values, and has an adjustment constraint for the MCL parameter set to a maximum value of 70 dB.

### Appendix C.3. DHAFES Rules, Inference, and Path Explanation

The Dynamic Hearing Aid Fitting Expert System (DHAFES) applies rules derived from standardized fitting guidelines, formulated in SWRL. These rules consider hearing aid settings, patient characteristics, and environmental factors to generate tailored fitting adjustments.

For instance, Rule 11 from the Balafon fitting guide (see Table 1) states, “If a patient reports that sound is too loud in a quiet environment, reduce the MCL parameter by 6 dB”. Equation (A1) formalizes this rule in SWRL:

(A1) $$\begin{array}{cc} \; & \mathrm{Patient}(?x)\wedge \mathrm{Hearing}\_\mathrm{Aid}(?ha)\wedge \mathrm{wearHA}(?x,?ha)\wedge \mathrm{Fitting}(?fit)\wedge \mathrm{hasFitting}(?ha,?fit)\wedge \\ \; & \mathrm{Complaint}(?com)\wedge \mathrm{doComplaint}(?x,?com)\wedge \\ \; & \mathrm{hasCause}(?com,?cau)\wedge \mathrm{hasEnvironnement}(?com,?env)\wedge \\ \; & \mathrm{swrlb}:\mathrm{equals}(?cau,141)\wedge \mathrm{swrlb}:\mathrm{equals}(?env,32)\Rightarrow \\ \; & \mathrm{has}\_\mathrm{fit}(?com,\mathrm{Action}\_1) \end{array}$$

Figure A3 illustrates this rule in action. The left panel (Figure A3a) shows patient PATX001 reporting a complaint: “In a quiet environment, the sound is too loud”. The right panel (Figure A3b) displays the system-generated adjustment, where Action_1 corresponds to lowering the MCL by 6 dB. The reduction by 6 dB comes from the results of the study carried out by Caswell-Midwinter and Whitmer [34] as stated in Section 2.3.

Beyond suggesting adjustments, DHAFES provides an explanation of its decision-making process, detailing the rules and conditions used. Table A2 presents this process. The system’s explanation can be summarized as follows:

“*I recommend reducing MCL by 6 dB because the patient experiences “loud sound” in a quiet environment. This suggests excessive amplification. Additionally, the patient may consider manually lowering the hearing aid volume*”.

Furthermore, the engine can specify which arguments are shared across multiple justifications and which are unique to a particular reasoning path. This allows for a clearer understanding of how different factors contribute to the final recommendation and whether they are consistently supported by other reasoning chains.

**Table A2:** Inference path and explanation.

#	Explanation	Justification
1	ComplaintX001_01 hasSpecificity 25	YES
2	ComplaintX001_01 hasCause 141	YES
3	doComplaint **Domain** Patient	NO
4	ComplaintX001_01 hasEnvironnement 35	YES
5	PATX001 doComplaint ComplaintX001_01	YES
6	hasCause(?com, ?cau), Patient(?p), equal(?env, 35), doComplaint(?p, ?com), hasSpecificity(?com, ?spec), equal(?cau, 141), hasEnvironnement(?com, ?env), equal(?spec, 25) ⇒ has_fit(?com, Action_1), hasRecommandation(?com, Baisser_Volume)	YES

## Appendix D. DHAFES—Fitting Platform

This section outlines how DHAFES operates, either as a standalone system or integrated with existing fitting platforms. Figure A4 illustrates the workflow: (1) The patient reports a complaint via a mobile questionnaire. (2) The encoded complaint, including patient details, hearing impairment, and current fitting, is sent to DHAFES via a specific API. (3) DHAFES processes the data, creates instances, and runs inference. (4) The system generates a fitting suggestion with recommendations and returns it to the fitting platform in JSON format. (5) The audiologist reviews the suggestion and either accepts (Figure A5a) or rejects (Figure A5b) it.
